# Exploring Intra and Interorganizational Integration Efforts Involving the Primary Care Sector – A Case Study from Ontario

**DOI:** 10.5334/ijic.5541

**Published:** 2022-09-08

**Authors:** Anum Irfan Khan, Jenine K. Harris, Jan Barnsley, Walter Wodchis

**Affiliations:** 1 University of Toronto, Canada; 2 Washington University in St Louis, USA

**Keywords:** network analysis, interorganizational collaboration, multimorbidity, primary care, case study

## Abstract

**Background:**

The primary care sector is uniquely positioned to lead the coordination of providers and organizations across health and social care sectors. This study explores whether **intra**organizational (professional) integration within a primary care team might be related to **inter**organizational integration between primary care and other community partners involved in caring for complex patients.

**Methods:**

Two care coordination initiatives (Health Links) were selected – one led by a primary care team with a high level of intraorganizational integration as assessed by the Collaborative Practice Assessment Tool (CPAT), and the other led by a primary care team with a low level of intraorganizational integration. A case study design involving a social network approach was used to assess interorganizational integration across six types of relationships including regular contact, perceived level of integration, referrals, information sharing, joint care planning, and shared resources.

**Results:**

Compared to the high-CPAT led case, the low-CPAT led case had higher density (more ties among organizations) in terms of regular contact, integration, and sharing of resources, whereas the opposite was true for the referral, information sharing, and joint care planning networks. Network centralization (extent to which network activity is influenced by one or a group of organizations) was higher for the high-CPAT case compared to the low-CPAT case in the integration, referrals, and joint care planning networks, while the low-CPAT case had higher centralization with regard to regular contact, information sharing, and shared resources.

**Conclusion:**

The interplay between intra and interorganizational integration remains unclear. We found no consistent differences in the patterns of ties across the six types of networks examined between the two cases. Assessing changes in network metrics for different organizations in each case over time, and supplementing network findings through in-depth interviews with network members are key next steps to consider.

## Introduction

As the prevalence of multimorbidity along with a rapidly aging population continues to present key challenges for health systems [1,2], adopting an integrated approach to enable collaboration between different levels of the health care system led by the primary care sector is increasingly critical to ensure that limited resources are effectively leveraged to optimize patient and provider outcomes [3,4,5,6,7]. The coordination of integration efforts within an organization, e.g., different disciplines within a primary care practice (intraorganizational integration), and across organizational boundaries (interorganizational integration) [8,9,10] are increasingly needed to more efficiently connect various levels of the health care system i.e., primary care, social supports, home care, and tertiary/acute care. In the context of primary care, intraorganizational integration can be conceptualized as professional integration or interprofessional collaboration within a defined organizational structure, which refers to collaboration between professionals based on shared competences, roles, responsibilities and accountability to deliver a comprehensive and coordinated continuum of care [11,12]. In contrast, interorganizational integration involves different organizations coordinating efforts to deliver comprehensive care over time, across professions and organizational boundaries to minimize fragmentation and enhance the value of services delivered [5,12,13,14,15].

Current literature indicates that there is a significant level of overlap in the factors that enable intraorganizational and interorganizational integration [16]. A commitment to a shared set of goals and accountability for outcomes, information technology capacity [17,18,19], and various aspects of interpersonal functioning such as trust and communication between team members, and leadership support for collaboration [20,21,22] represent common facilitators that enable both intra and interorganizational integration [16]. Differences in professional culture across disciplines and organizations, gaps in role clarity, the absence of shared goals and values, and operational disconnects, i.e., variability in budgetary and planning cycles [23,24,25] are among the key challenges organizations face in collaborating effectively both within and across organizational boundaries [24,26,27,10,28,16,29]. While there has been growing emphasis on the adoption of principles for integrated care as part of health system reform in Canada and globally, to date, our understanding of the interplay between intra and interorganizational integration is limited.

In the context of Ontario, an effort to improve the health system’s capacity to better care for complex patient populations, defined as individuals with multiple chronic conditions, functional and/or cognitive impairments, and social vulnerability [30,31] has resulted in various initiatives to better integrate health and social care for this patient population. This includes the advent of interprofessional primary care teams, as well as formal interorganizational partnerships between networks of providers and organizations across sectors through the Health Links program [32,33,34,35]. The Health Links initiative involved formalizing existing geographic networks of providers including primary care practices, hospitals, long-term care and community agencies, to support interorganizational integration via a shared commitment to coordinate care across sectors for complex high-cost patient populations [36]. But despite strategic and policy efforts to promote integrated care for complex patient populations in Ontario, and emerging evidence around the shared facilitators for intra and interorganizational integration [16], our understanding of the extent of interorganizational integration, and the interplay between intra and interorganizational integration is still in its early stages. Given this gap in knowledge, the objective of this study was to examine whether intraorganizational (professional) integration within a multidisicplinary primary care team is associated with interorganizational integration between the primary care sector and other types of organizations involved in delivering services for complex patient populations, as health and social care systems adapt to changing disease burdens and patient needs.

## Methods

### Setting and participants

This setting for this study was Ontario (Canada), which has a publicly funded healthcare system that offers universal health care coverage for physician and hospital services. The primary care sector serves as a gateway to the health system, and includes a range of funding arrangements including salaried models, blended fee-for-service and blended capitation. Over the past decade, there has been a transition away from traditional fee-for-service payment models, as part of a systematic shift towards interprofessional models of team-based primary care including Community Health Centres (CHCs) and Family Health Teams (FHTs), in concurrence with a strong emphasis on collaboration between primary care and other providers/organizations in the broader health and social care landscape, which informed the establishment of the Health Links program in 2012 [37]. The objective of the Health Links initiative was to promote coordination between providers and organizations to better serve complex patient populations, defined as patients with four or more chronic conditions, including a focus on individuals living with mental health and addictions, and the frail elderly, to ultimately help reduce healthcare utilization (e.g., emergency department visits, hospitalizations, and readmissions) and improve access to primary and specialist care [32,34,36,38].

By design, all organizations in a Health Link work collaboratively to develop a care plan for the patient and deliver coordinated care with the patient at the center [39]. Each Health Link serves a catchment area with a total population of at least 50,000 people, and involves engagement from at minimum 65% of primary care providers in a given geographic area [32]. At the time of this study, 82 Health Links were operational across Ontario with an estimated 1800 partner organizations from multiple sectors [34].

Each Health Link is led by a lead agency, which could be a primary care practice, hospital, or home and community support agency [32,40]. The lead agency is required to take ownership of operational, management and agenda-setting aspects for each Health Link [32,40]. To allow for an in-depth examination of the possible interplay between interprofessional collaboration within primary care practices is associated with interorganizational collaboration, two Health Links led by interprofessional primary care teams that had previously participated in a survey of interprofessional collaboration [41], were selected as case studies. The use of a case study design was selected to allow for an in-depth multi-faceted exploration of how organizations were collaborating within the boundaries of a Health Link [42].

Intraorganizational (professional) integration within each lead agency was measured using the Collaborative Practice Assessment Tool (CPAT) [43]. A recent review of instruments to assess interprofessional collaboration found that the CPAT is considered to be the most appropriate instrument to measure collaborative practice involving providers from a broad range of disciplines, particularly as it relates to chronic disease management [44]. The tool has been used by providers across different types of healthcare settings including primary care teams [43,45]. The CPAT is comprised of 56 questions across a total of 8 domains, and is scored by averaging all items within each domain, and a total measure of professional integration is generated by adding up the average domain scores [46]. The CPAT was administered to individual providers within each lead agency (interprofessional primary care team) for the two Health Links. Individual provider-level scores are then aggregated to develop a team-level CPAT score. The lowest possible total score on the CPAT is an 8, whereas the highest possible score is a 56.

Interorganizational integration in each Health Link was explored through a social network analysis [15], which is increasingly being used in health services research to evaluate integrated care programs/services [47,48,49]. Assessing linkages between organizations using a network lens is grounded in the idea that service systems can be conceptualized as ‘networks’ of interacting organizations, and corresponding network characteristics (i.e., connectivity and flow of information between members in the network) can be inferred from the number and patterns of relationships between those organizations [50,51,52].

### Case selection

The cases for this study were based on a larger study of 66 primary care teams that completed the CPAT survey [41]. The mean CPAT score across participating practices was 46.6 (sd = 2.47); the small standard deviation revealed a limited spread of scores given the possible range of 0 to 56. Of the 66 primary care teams that participated in the original study, 17 primary care teams were involved in the Health Links program, and ultimately two Health Links led by primary care teams were selected to serve as case studies based on their CPAT scores.

To capture the influence of variability in intraorganizational integration (as assessed by the CPAT scores) on interorganizational integration, the first Health Link selected to serve as a case was led by a primary care team with a low CPAT score [43.74], hereby referred to as the ‘low-CPAT led case’, whereas the second case was led by a primary care team with a high CPAT score [50.01] thus referred to as the ‘high-CPAT led case’. Both Health Links, were led by an interprofessional primary care team (which had a broad range of interdisciplinary providers including family physicians, registered nurses, social workers, and dieticians etc.), and included hospitals, a home and community service agency responsible for coordinating community-based care, a long-term care facility. Overall, the two cases were comparable in terms of the number, types health and social care providers involved, years in operation, geographic area served, and population demographic features (age, medical and social complexity).

### Data Collection

A network questionnaire comprised of 6 questions was developed based on by past studies involving interorganizational network analyses, and adapted to reflect the mandate of the Health Links program [53]. The questionnaire sought to explore six types of relationships including: frequency of contact (daily, weekly, monthly, bi-annually, and annually) between organizations perceptions around the level of integration between organizations via a scale ranging from not linked to fully linked (see Table 1) [53,54,55], referrals, information sharing, joint care planning, and shared resources. In addition to data collected for the social network analysis, a general question around network member views on the benefits and drawbacks of their involvement in the Health Links program was also included in the network questionnaire (see Appendix 1).

**Table 1:** Response options for perceived levels of integration between organizations.


LEVEL OF INTEGRATION	DESCRIPTION

**Not linked**	We did not work together (to serve patients with multiple chronic conditions) at all and have separate program goals

**Communication**	We shared patient information only when it was advantageous to either or both programs

**Cooperation**	We shared patient information and worked together when an opportunity arose

**Coordination**	We worked side-by-side as separate organizations to achieve common program goals; efforts were coordinated to prevent overlap

**Collaboration**	We worked side-by-side and actively pursued opportunities to work together to support patients with multiple chronic conditions, but did not establish a formal agreement

**Partnership**	We worked together as a formal team with specified responsibilities to achieve common goals (had a Memorandum of Understanding or other formal agreement)

**Fully linked**	We mutually planned and shared staff and/or resources to organize and deliver care for individuals with multimorbidity


Given the central role that the lead agency plays in managing the Health Link, a list of organizations conceived to be a part of the Health Link was then shared with the executive director of the lead agency for each case to get their feedback on the appropriateness of organizations considered for inclusion in the network analysis, and determine which individual from each organization would be best suited to complete the network questionnaire. An electronic link to the network questionnaire was sent to the individual identified as the contact person for each organization in the Health Link. All data was collected between May and July 2018 through the Hosted in Canada Survey Platform. Responses were stored on a password-protected server, which only the research team was able to access. Ethics approval was obtained from the Research Ethics Board at the University of Toronto (Protocol ID 33699).

### Data analysis

Data from the Hosted in Canada Survey Platform was imported into UCINET to compute network measures and generate visualizations. Network plots were developed to illustrate the structure and functioning of each case as it relates to the six types of networks that were explored. A detailed description of the network metrics generated is available in Table 2. Missing data were imputed using reconstruction for undirected networks (where there is no specified direction of the tie) [56]. For each relationship between two nodes in an undirected network, when only Organization A and Organization B answered (not both), reconstruction uses the answer of the single node as the relationship value. So, if only Organization A answered the survey and reported a tie with Organization B, the network data would assume that Organization A and Organization B are connected [57].

**Table 2:** Description of network and node level measures.


MEASURE	DESCRIPTION

**Density**	A network-level metric that represents the overall level of connectedness among organizations in the network, based on the proportion of actual links relative to the maximum number of possible links in the network [55]. Density scores range from 0 to 1 – lower scores indicate low levels of connectedness, i.e. a 0 would entail no connections between members in the network, whereas a 1 means that all network members are connected to one another [15].

**Degree centralization**	A network-level measure that depicts how centralized a network is overall, i.e., the extent to which network activity is influenced by one or group of organizations based on the number of connections each network member has with others [54,60,61]. Degree centralization ranges from 0 to 1 – a higher value of degree centralization indicates that a small number or group of organizations have a major influence on network activity.

**Degree centrality**	Degree centrality is a node-level measure and typically denotes the number of links each member has with other members in the network – organizations with high degree centrality are considered to be well connected within the network [62]. For directed networks, there are two possible measures of degree centrality: (1) *in-degree centrality* represents the number of incoming ties to a particular member, and (2) *out-degree centrality* reflects the number of outgoing ties [63]


The contact measure was dichotomized based on a cut-off at biannual contact, so organizations that had contact with each other more than twice per year were considered linked, while those with less contact than this were considered not linked. In the event of a discrepancy between members in a dyad, the average value was used [53]. The contact network was considered to be undirected in nature, and since missing data was below 30%, reconstruction was used to impute those responses where applicable [58].

Participant views on the perceived level of integration between organizations were dichotomized; organizations were considered to be integrated if they indicated the nature of their relationship as ‘coordination’ or above (coordination, collaboration, partnership, or fully linked) [53]. Mean tie value was used in the event of a discrepancy between two organizations on the perceived level of integration with other network members.

The referral network included both sent and received referrals. Only confirmed ties were analyzed A line between two nodes (organizations) indicates the presence of a confirmed referral relationship; i.e., Organization A indicated they sent a referral to Organization B, and Organization B expressed that they have received a referral from Organization A. Density and degree centralization scores under 0.30 were considered to be low, 0.30–0.50 were seen as moderate, and above 0.50 was deemed high [59].

## Results

There were 15 organizations in the low-CPAT led case, and 12 organizations in the high-CPAT led case. Both cases had a very high response rate – 93.3% for the low-CPAT led case (n = 14/15), and 91.7% for the high-CPAT led case (n = 11/12). A 75% response rate is considered sufficient in terms of reliability for network data [64,65].

### Network measures and visualizations

**Figure 1:**
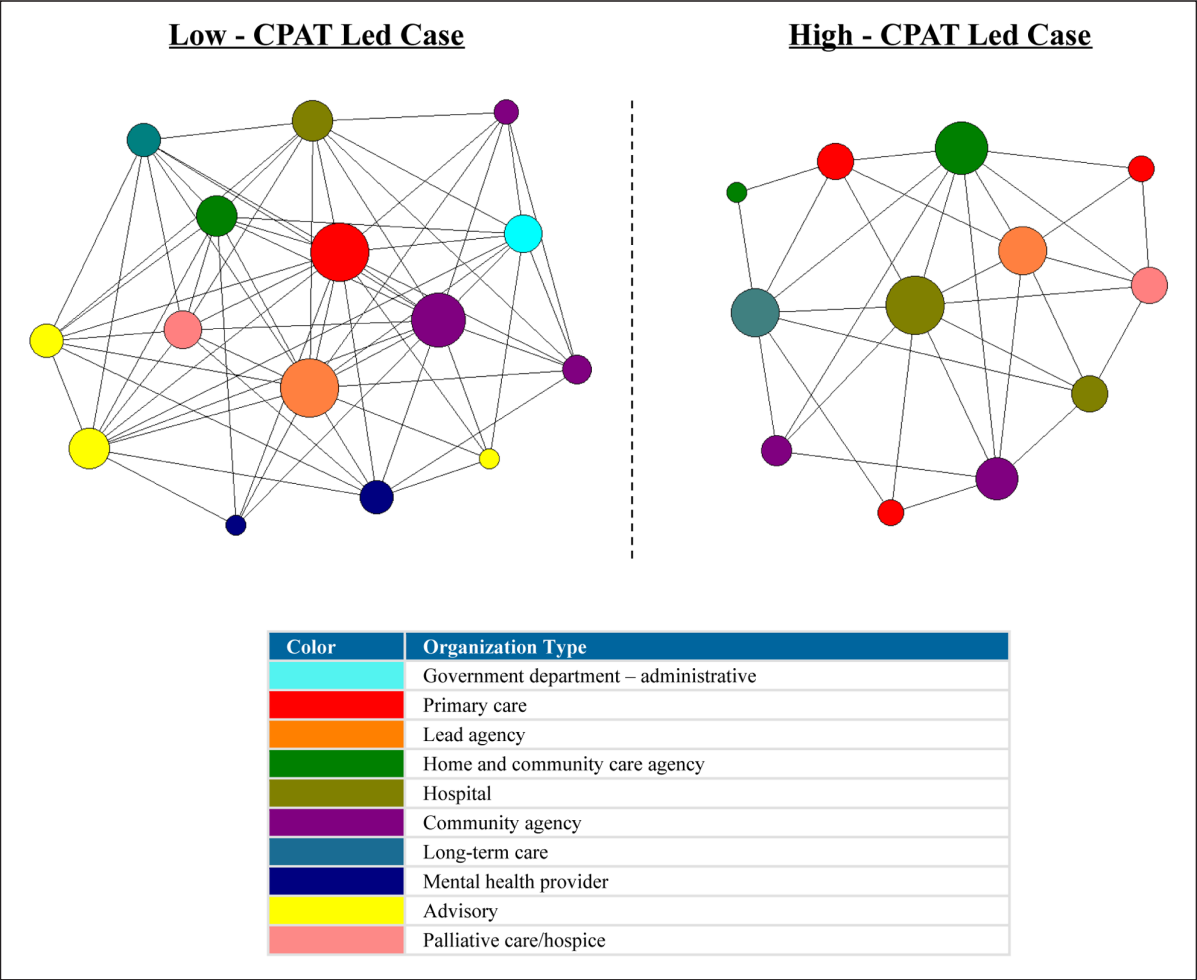
Regular contact (monthly or more often) between organizations in two Health Links in Ontario – node size depicts degree centrality for each network member (data collected in 2018).

**Figure 2:**
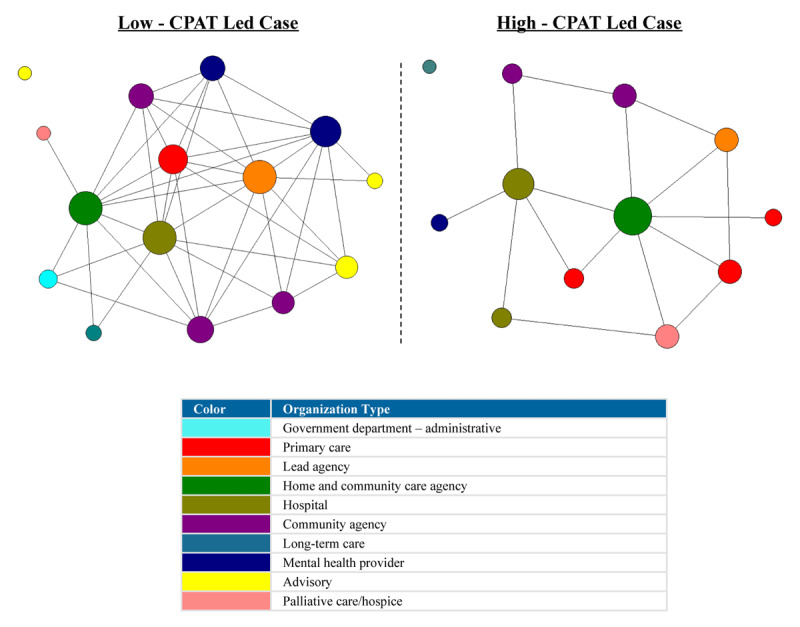
Perceived level of integration (at minimum the presence of a coordinated relationship) between organizations across two Health Links in Ontario – node size depicts degree centrality for each network member (data collected in 2018).

**Figure 3:**
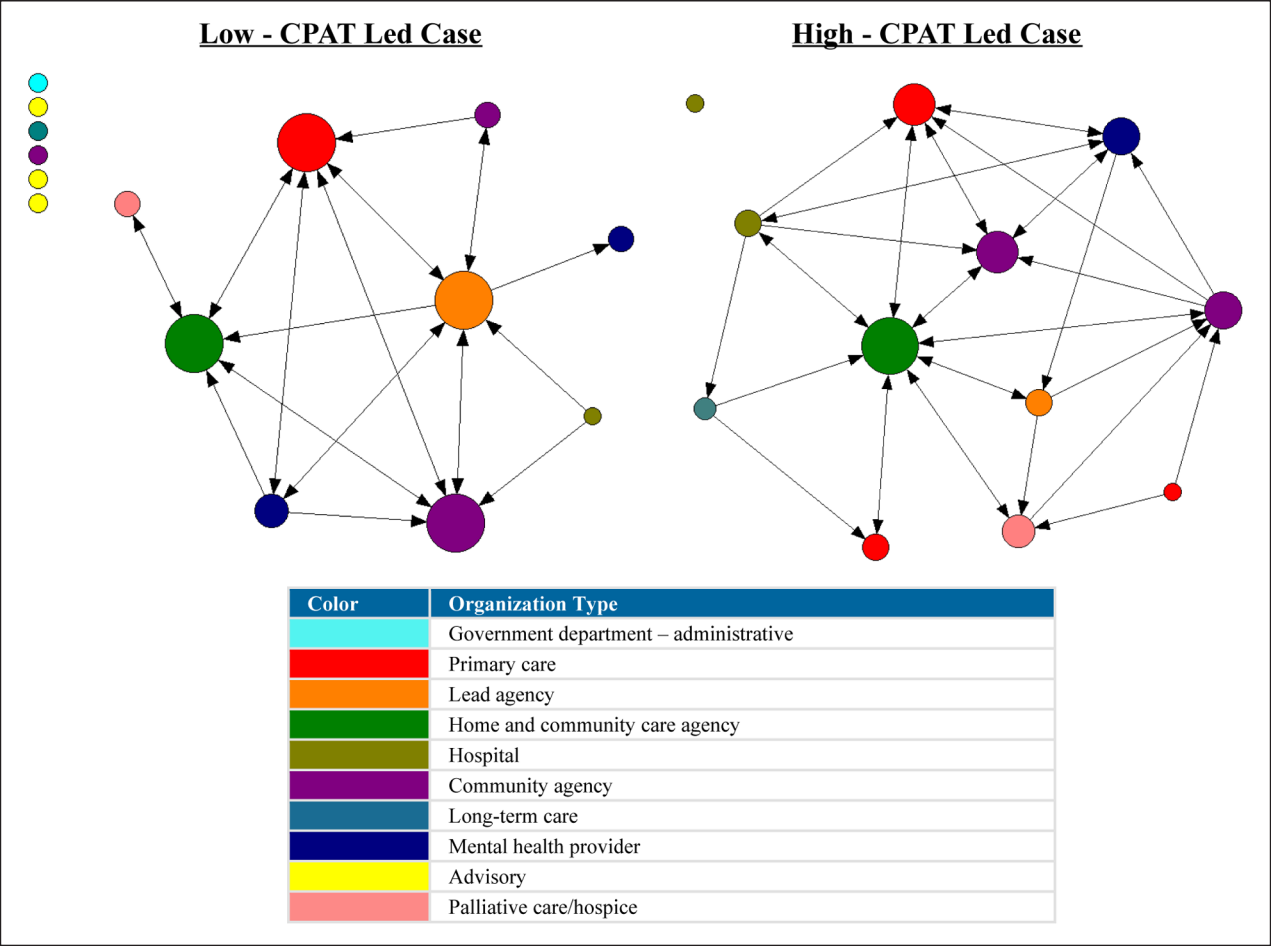
Referral relationships between organizations in two Health Links in Ontario, Canada–node size depicts in-degree centrality for each organization, and the arrowhead reflects the direction of the referral (i.e., being sent or received) (data collected in 2018).

**Figure 4:**
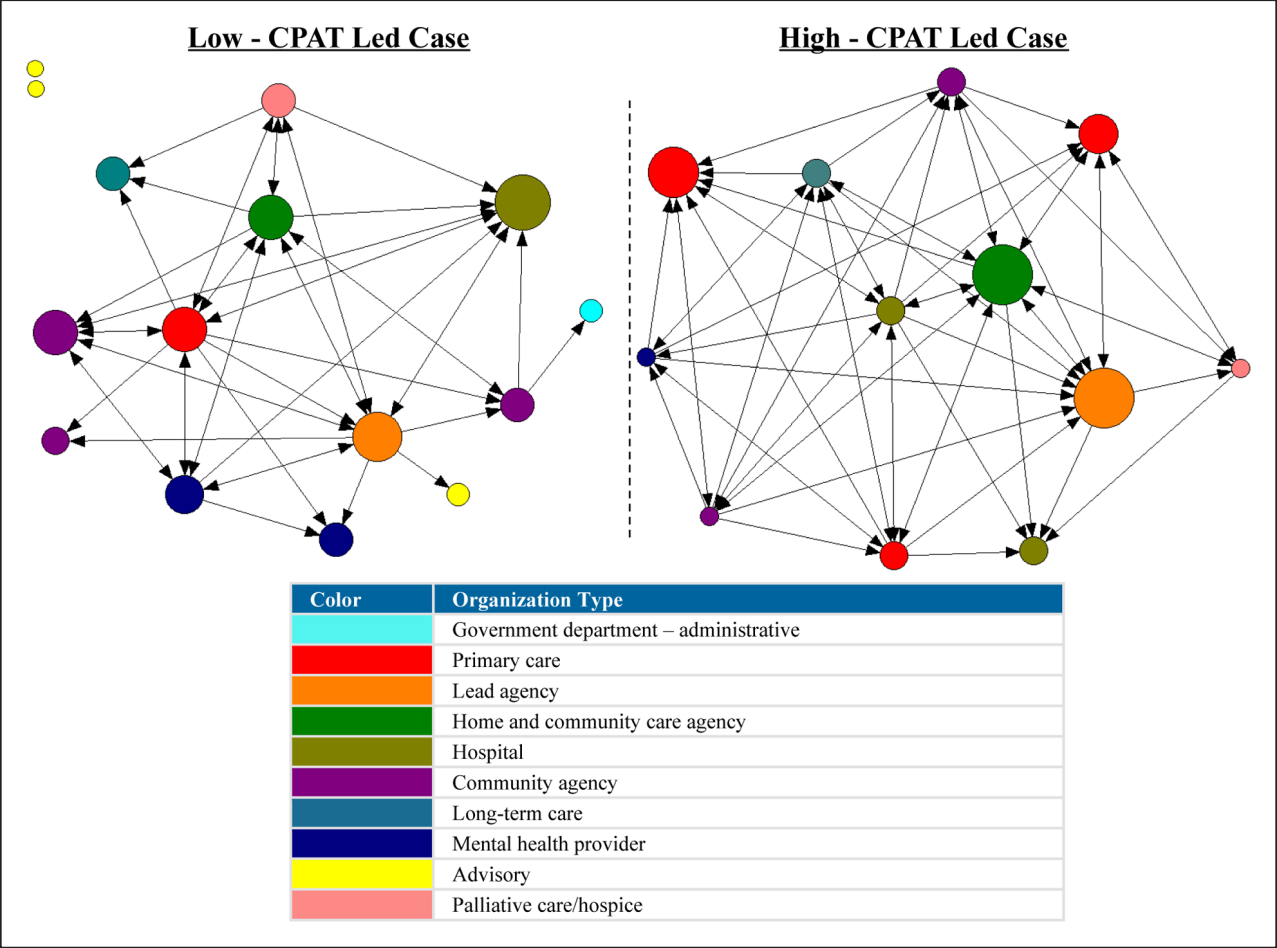
Information sharing relationships between organizations in two Health Links in Ontario – node size depicts in-degree centrality for each organization, and the arrowhead indicates the direction of information sharing between network members (data collected in 2018).

**Figure 5:**
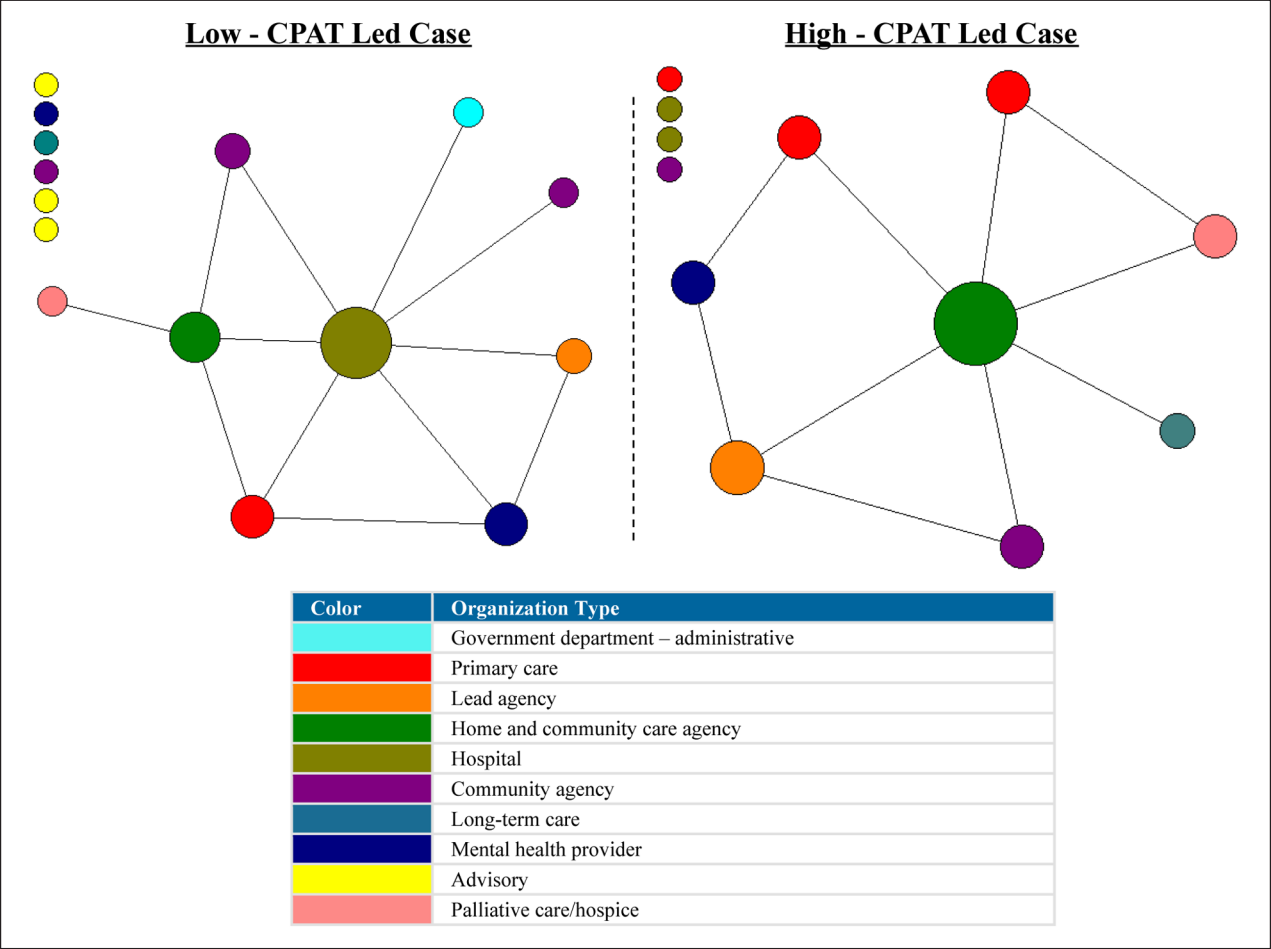
Joint care planning relationships between organizations in two Health Links in Ontario – node size depicts in-degree centrality for each network member (data collected in 2018).

**Figure 6:**
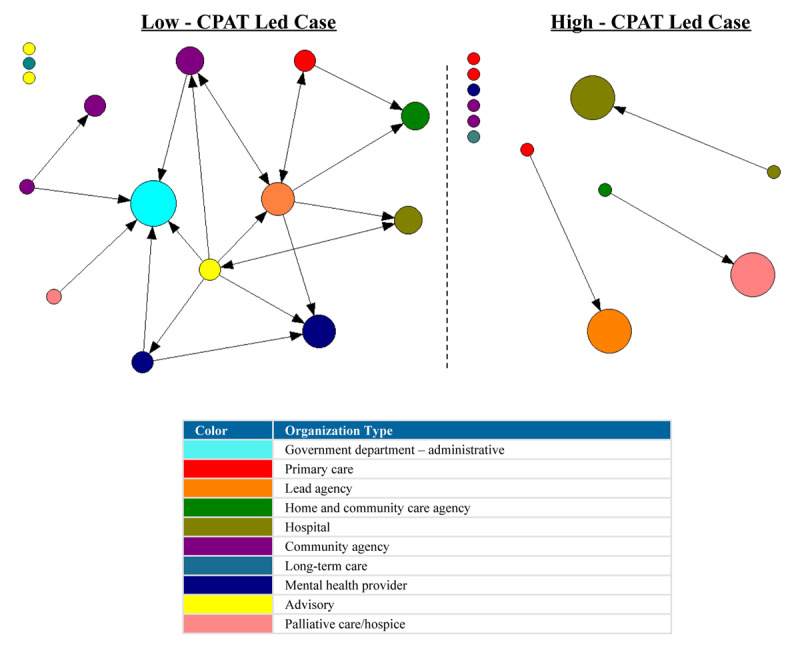
Resource sharing relationships between organizations in two Health Links in Ontario – node size depicts in-degree centrality for each organization, and the arrowhead indicates the direction of resource transfer between network members (data collected in 2018).

For the network plots in Figures 1, 2, 3, 4, 5, 6, the nodes represent each organization in the network and the color of a node depicts the type of organization, i.e., lead agency, hospital or community agency. The size of individual nodes in each network map indicates degree centrality (number of connections each organization has with other network members) in undirected networks, and in-degree centrality (the number of incoming ties an organization has) in directed networks (referrals, information sharing, joint care planning and shared resources), wherein larger nodes represent organizations that are more prominent in terms of connectivity and play a central role in the network. A summary of the network measures generated across the 6 networks is available in Appendix 2.

#### Contact

For the contact network, the low-CPAT led case showed a high density score, compared to a moderate level of density in the high-CPAT led case, revealing that 64.8% of linked dyads (pairs of connected organizations) in the low-CPAT led case had regular contact compared to 48.5% in the high-CPAT led case. Node size in Figure 1 represents degree centrality (number of links each member has with other members in the network), and a line connecting two nodes (organizations) indicates regular contact (daily, weekly or monthly basis). Both cases showed a moderate level of degree centralization, but the structural positioning of network members varied considerably across cases. Primary care played a central role in the contact network for the low-CPAT led case, with the lead agency and another primary care practice exhibiting the highest levels of degree centrality, compared to a hospital that had the highest degree centrality in the high-CPAT case.

#### Perceived level of integration

The low-CPAT led case had a moderate density score (0.400) compared to the low-density score observed in the high-CPAT led case (0.242). A total of 40% of possible linked dyads (pairs of nodes/organizations) in the low-CPAT led case indicated being engaged in at minimum a coordinated relationship with other organizations in the network, compared to 24% in the high-CPAT led case. Node size in Figure 2 represents degree centrality, and a line between two nodes indicates that both organizations reported having *at minimum* a coordinated relationship, i.e., organizations worked side-by-side as separate organizations to achieve common program goals, and efforts were coordinated to prevent overlap. In the low-CPAT led case, the lead agency (a primary care practice), and a hospital had the highest degree centrality, showcasing their prominent positioning in the network. For the high-CPAT led case, a government agency that coordinates home and community care services had the highest degree centrality, revealing its central position in the network.

#### Referrals

The density score of the high-CPAT led case revealed a moderate level of connectivity in terms of referral relationships compared to a low level of connectivity in the low-CPAT led case. A total of 37.9% of possible linked dyads (pairs of nodes/organizations) in the high-CPAT led case were involved in referral relationships, compared to only 11.9% in the low-CPAT led case. Referral relationships in both cases were moderately centralized around a few organizations. The arrowhead in Figure 3 indicates the direction of the referral, i.e., whether the referral was being sent or received by any given node/organization (Organization A sent referral → Organization B received referral). Figure 3 reveal several isolates in the low-CPAT led case; 6 of the 15 organizations in this Health Link were not engaged in any type of referral relationships, compared to 1 of 12 organizations in the high-CPAT led case. Node size reflects in-degree centrality (number of incoming referral ties) for each organization.

#### Information sharing

A low density score (0.229) was observed in the low-CPAT led case, indicating limited connectivity in terms of information sharing relationships between network members in this case, compared to the moderate density score (0.500) observed in the high-CPAT led case, as observed in Figure 4. A total of 22.9% of possible linked dyads (pairs of nodes/organizations) in the low-CPAT led case were involved in information sharing relationships, compared to 50% in the high-CPAT led case. The size of each node in Figure 4 represents in-degree centrality (number of incoming information sharing ties), and the arrowhead indicates the direction of information sharing. Information sharing in the low-CPAT led case was centered around the primary care sector; the lead agency (a primary care practice) and another primary care practice had the highest out-degree centrality (outgoing information sharing ties), and a hospital had the highest in-degree centrality. In the high-CPAT led case, the home and community care agency and a hospital had the highest out-degree centrality whereas the same home and community care agency along with the lead agency had the highest in-degree centrality.

#### Joint care planning

Both cases had low density and moderate degree centralization in the joint care planning network. Only 11% of possible linked dyads (pairs of organizations) existed in the low-CPAT led case, compared to 15% in the high-CPAT led case. A line connecting two nodes in Figure 5 indicates that presence of a confirmed joint care planning relationship and node size represents degree centrality for each organization (number of joint care planning relationships with other organizations in the Health Link). Joint care planning in the low-CPAT led case was led by a hospital, which had the highest in-degree and out-degree centrality, compared to the high-CPAT led case, where joint care planning efforts were led by the home and community care agency. Both networks were very centralized as demonstrated by the large central node. Trends in network metrics and node-level measures were consistent when unconfirmed ties were analyzed.

#### Shared resources

The shared resources network had the lowest density scores across the 4 types of relationships explored through the network analysis. A total of 10% of possible linked dyads (pairs of nodes/organizations) were involved in resource sharing relationships (e.g., providing funding for shared staff members) observed in the low-CPAT led case, compared with only 2.3% in the high-CPAT led case. Node size in Figure 6 represents in-degree centrality (number of links depicting incoming resources) for organizations in each Health Link. The arrowhead indicates the direction of resource sharing, i.e., which organization is providing a given resource and which organization is on the receiving end (e.g., Organization A is sending resources → Organization B receiving resources). The lead agency for the low-CPAT led case had high in-degree and out-degree centrality indicating that it receives and offers resources to other organizations in the Health Link. A total of 12 (out of a possible 15) organizations in the low-CPAT led case were involved in resource sharing relationships, compared to only 6 organizations in the high-CPAT led case were involved in resource sharing relationships, and several isolates are visible in Figure 6.

### Perceived benefits and drawbacks

Respondents were asked to offer feedback on perceived benefits and drawbacks pertaining to interorganizational integration in each Health Link. Network members may have varying expectations at the outset of a partnership being established [24,66], so developing a baseline understanding of member perspectives around whether expectations have been met, and what challenges they face, can help facilitate improvements in network structure and functioning [66]. A majority of respondents in both cases indicated that being involved in Health Links, had resulted in or was expected to support improvements in their capacity to better serve patients in their communities, and improve the use of each organization’s services, illustrating that some of the benefits intended in the development of the Health Links initiative have been realized.

There was a clear difference between the two cases with regards to perceived drawbacks. A total of 18% of network members in the high-CPAT led case, stated a loss of control/autonomy over decision-making, and 36% of network members felt that involvement in the Health Link had or is expected to cause strained relations within their organization. In comparison, none of the members from the low-CPAT led case felt that their engagement in these interorganizational partnerships resulted in a loss of control/autonomy over decisions or strained relations within their organization. Only 14% of organizations in the low-CPAT led case believe their involvement in Health Links does or is expected to take too much time and resources, compared to 45% of network members in the high-CPAT led case. A summary of participant feedback on perceived benefits and drawbacks is available in Appendix 3.

## Discussion

To date little is known about the extent to which integration efforts within an organization may influence interorganizational integration. The purpose of this study was to explore intra-organizational integration (professional integration) within a primary care team that leads a Health Link, is associated with interorganizational integration between primary care and other organizations involved in delivering services within the Health Link.

For the contact network, the low-CPAT led case had higher density than the high-CPAT led case, indicating that there were more contact ties between organizations in the low-CPAT led case compared to the high-CPAT led case. Greater density in a network signals the presence of more pathways among organizations, that facilitate communication and connectivity between network members, compared to less dense networks [61]. Past research has found that higher density scores are indicative of trust between organizations, greater social capital, and a commitment to engage in achieving shared goals and objectives [67,68]. Higher network density can also reflect greater homogeneity in the network; individuals or groups are more likely to associate, accept and bond with others that share similar characteristics and/or beliefs [69], which can facilitate a higher likelihood of information exchange and collaboration between network members [70].

Regular contact between organizations in the low-CPAT led case may also explain the higher density score for this Health Link in the integration network; a total of 40% of possible linked dyads (pairs of organizations) in the low-CPAT led case were involved in at minimum coordinated relationships, compared to only 24% in the high-CPAT led case. While both cases had a similar number and mix of organizations, overall, for the low-CPAT case the primary care sector appeared to play a crucial role in connecting organizations that would not otherwise be in contact. In contrast, for the high-CPAT led case, the home and community care service agency, and a hospital had the highest degree centrality, highlighting the less prominent role of the primary care sector in this Health Link.

Mixed trends were observed in terms of network measures that were assessed. The low-CPAT led case appears to have higher density in the contact, shared resources, and perceived level of integration networks, compared to the high-CPAT led case. In contrast, the high-CPAT led case has higher density compared to the low-CPAT led case with regards to sending and receiving referrals, information sharing, and joint care planning. Past studies report associations between regular contact and information exchange or ‘diffusion’ across a network [71,72]. The low-CPAT led case had high density for contact and moderate density in the integration network and so could be expected to have strong connectivity across the four other types of relationships [71], however our findings indicated otherwise. This could be attributed in part due to strong informal communication between organizations in the low-CPAT case, which can precipitate information sharing and referrals through informal mechanisms (e.g., ad-hoc communication, leveraging existing relationships to call colleagues in another organization to discuss a patient’s needs etc.) [73,74,75]. A higher degree of direct contact between network members, may have led organizations in low-CPAT led case to have greater awareness of the types of resources available in the Health Link, and thus directly coordinate care for patients through their frequent contact (possibly resulting in fewer referrals). A recent examination of interagency partnerships between health and social care organizations in the UK also found that coordination between agencies was found to rely more on informal rather than formal relationships [76]. Compared to the low-CPAT led case, the high-CPAT led case had lower density in the contact and integration networks, but higher density in a majority of the relationship networks – illustrating that most of the collaboration between organizations in this case were occurring through formal mechanisms. This finding aligns with past research, which found that lower density in networks corresponds with more formal collaboration or interaction between network members [77].

Degree centralization was similar between cases across all networks, except for the information sharing and referrals networks, wherein the high-CPAT led case had higher degree centralization scores compared to the low-CPAT led case. Greater centralization in a network reflects the presence of ‘hubs’ of network members, who given their central positioning can transmit information to other members in an efficient manner [61]. This could also have contributed in part to the higher density observed in both the information sharing and referrals network for the high-CPAT led case, compared to the low-CPAT led case.

Network metrics reveal that in the high-CPAT led case, organizations appear to be functioning more independently (fewer sharing of resources between members) and have left the coordination of integration between network members to an organization (a home and community care agency) that is not the lead agency (a primary care practice). Whereas in the low-CPAT led case, organizations contact each other more regularly (though fewer referrals are sent between organizations), potentially facilitating a higher perception of interorganizational integration between members in this Health Link, which also features strong involvement by the lead agency (a primary care practice) in many of the key relationships examined.

Past research has shown that the lead agency for a coalition or program typically wields considerable influence in the network based on their structural positioning [53,71,72]. Network findings indicate that the lead agency in the low-CPAT led case, had the highest degree centrality for the contact and integration networks, and well as high in-degree and out-degree centrality in all but the one of individual relationship networks examined (information sharing, referrals, and shared resources). Whereas in the high-CPAT led case, the lead agency did not hold the highest in-degree and out-degree centrality in a majority of the networks examined. This variation in structural positioning of the lead agency between the two cases indicates possible differences in the ‘embeddedness’ (assessed by total degree, in-degree and out-degree centrality) each lead agency exhibited in their respective Health Link [78]. Organizations with a low level of embeddedness are considered less prominent in terms of their reputation, less likely to be trusted by other network members, and would typically perceive less overall benefit from their participation in a given network [78]. While direct contact with an influential node can facilitate the adoption of a given behavior or innovation [79], the lead agency in the high-CPAT led case was not prominent in terms of its structural positioning for most of the 6 networks explored, thus potentially restricting the extent to which diffusion of innovation between intraorganizational and interorganizational collaboration, could have occurred.

A majority of network members across cases reported a number of benefits associated with their involvement in the Health Link, including improvements in their capacity to care for patients with multimorbidity, and better use of their organization’s services. However, a greater proportion of network members in the high-CPAT led case felt there were drawbacks to their engagement in Health Links; whereas none of the respondents in the low-CPAT led case reported difficulty in dealing with other members in the network, while 27% of network members in the high-CPAT led case indicated that this had already occurred, and 18% of respondents in the high-CPAT led case (but none of the members in the low-CPAT led case), felt their participation in Health Links had resulted in a loss of autonomy. This contrast between the two cases is reflected in some of the network metrics. Specifically organizations in the low-CPAT led Health Link were more likely to participate in resource sharing relationships, and reported overall a higher degree of perceived integration than those the high-CPAT led Health Link, resulting in perhaps a more effective distribution of roles and responsibilities between network members, in turn reducing possible burnout from their involvement in the Health Links program. The limited influence of the lead agency in the high-CPAT led case may have affected the overall cohesiveness of the Health Link, possibly contributing to lower perceived levels of integration, tension or burn-out among network members [80,81], and a more negative outlook on the experience of participating in this interorganizational integration initiative.

### Strengths

This study offers a unique assessment of how organizations across the health and social care sectors in Ontario are engaged in integrating care across organizational boundaries, which is increasingly critical in improving the capacity of the health system to better respond to changing disease burdens and evolving patient needs [82]. The Health Links program was designed to be a network intervention intended to enable structurally and functionally distinct organizations to collaborate under a shared mandate to reduce fragmentation, minimize redundancies [28,40], and ultimately improve patient and system-level outcomes [83]. This study offers an in-depth look at how organizations in two Health Links are collaborating to deliver care across organizational boundaries, and provides a starting point to explore the interplay between different types of integration efforts using an innovative network lens. A systematic approach involving in-depth stakeholder consultation was used to determine network boundaries, and a wide range of relationships between organizations were examined to provide a fulsome sense of the mechanisms through which organizations are working together to deliver an integrated continuum of care. Both cases had a high response rate to the network questionnaire; network data included in the analysis included representation from over 90% of organizations in each Health Link. Both confirmed and unconfirmed ties were analyzed to identify consistency in trends across the 6 networks assessed, and trends across network metrics and node-level measures were consistent when only confirmed ties were included in the analysis.

### Limitations

Several important limitations must be noted in interpreting these findings. In terms of external validity, given the small number of cases, findings may not be applicable to other jurisdictions or types of interorganizational partnerships. Due to the small sample size and cross-sectional study design, a longitudinal comparison of trends in network density and centrality measures could not be conducted. Given the small number of cases involved, it was not feasible to examine the impact of this interorganizational integration initiative on individual or system-level outcomes for patients served by Health Links. Furthermore, while theoretical overlap in the drivers of intraorganizational and interorganizational integration has been previously explored [16], a measure of interprofessional collaboration was only available for the lead agency in each of the case studies, rather than for every organization in the Health Link, thereby restricting our ability to empirically explore the relationship between intraorganizational and interorganizational integration.

## Conclusion and Recommendations

Given the absence of a clear trend across the 6 networks assessed for each case, the interplay between interprofessional and interorganizational integration remains unclear. However both cases indicated perceived improvements in their capacity to care for patients with multiple chronic conditions through access to a wider range of providers and coordination between participating organizations, and better use of their organization’s services through their participation in the Health Links initiative. In terms of network structure the role of the lead agency (a primary care team) plays an important role in influencing network dynamics; and ensuring that the lead agency is set up to model best practices (i.e., regular contact, sharing of resources with other network members etc.) and promote the adoption (or non-adoption) of behaviors for other network members. Results suggest ensuring that the designated lead agency is adequately positioned to influence network functioning, and that organizations with prominent structural positioning (high degree centrality) should be leveraged to disseminate best practices and better optimize for interorganizational collaboration at a provincial scale.

Continuous and on-going interaction between network members plays an important role in building trust as well as social capital, by shaping patterns of interaction over time [67,84], thus capturing the influence of temporality on network functioning is highly recommended [15,24,49,66]. As such assessing changes in the roles and prominence of different organizations over time can allow providers, policymakers and leadership to adapt resources, structures and/or supports for organizations involved in interorganizational partnerships. And while the use of a network analysis approach to understand the structure of each network is an important first step in exploring how Health Links are operating, a more detailed assessment through interviews or focus groups with organizations in each Health Link, may be helpful to further identify and contextualize the factors that may inform integration efforts in each case. In terms of next steps, incorporating measures of intraorganizational (professional) integration for all network members (instead of the lead agency only), and an evaluation of the impact of network functioning on key patient and system-level outcomes are important next steps to consider.

## Additional Files

The additional files for this article can be found as follows:

10.5334/ijic.5541.s1 Appendix 1. Network Analysis Questionnaire.

10.5334/ijic.5541.s2 Appendix 2. Summary of network measures for two Health Links in Ontario (data collected in 2018).

10.5334/ijic.5541.s3 Appendix 3. Member perceptions around the benefits and drawbacks of involvement in the Health Links program.
