# Supplementary material for: Exploring Intra and Interorganizational Integration Efforts Involving the Primary Care Sector – A Case Study from Ontario
Source: Int J Integr Care. 2022 Sep 8;22(3):15. doi: 10.5334/ijic.5541 (PMC9461681; doi:10.5334/ijic.5541)
Supplement: Appendix 1. — Network Analysis Questionnaire. [file ijic-22-3-5541-s1.pdf]

**Appendix 1: Network Analysis Questionnaire**

**Overview:**
You are invited to participate in this study titled, ‘Managing Multi-Morbidity In Primary Care: Examining integration efforts within and across boundaries’, being conducted by researchers at the University of Toronto. Your organization has been identified as being a member of a broader network of organizations involved in delivering services to adults with multi-morbidity in this community.

**Research objectives:**
This study seeks to explore how organizations collaborate across organizational boundaries (inter-organizational integration) to serve adults with multi-morbidity. This collaboration may include shared decision-making around patient care/treatment, exchanging patient information, or simply referring patients for treatment and/or programs etc. We are adopting a network analysis approach to examine four aspects of integration across organizational boundaries:

- **A. Frequency of contact**
- **B. Types of links between organizations**
- **C. Relationship Quality Benefits**
- **D. Drawbacks of integration across organizational boundaries**

1. **Please select the organization you represent:**
   - ☒ Organization A
   - ☐ Organization B
   - ☐ Organization C
   - ☐ Organization D
   - ☐ Organization E
   - ☐ Organization F
   - ☐ Other, please describe _______________

2. **Contact: In the past year, on average, how often did you have contact with the following organizations - contact can be meetings, phone calls, or emails.**

| Network members | Never | Annual | Biannual | Quarterly | Monthly | Weekly | Daily |
|---|---|---|---|---|---|---|---|
| **Organization A** | | | | | | | |
| **Organization B** | | | | | | | |
| **Organization C** | | | | | | | |
| **Organization D** | | | | | | | |
| **Organization E** | | | | | | | |
| **Organization F** | | | | | | | |

3. **Link Type: How would you describe the type of relationship your organization has with the following organizations in the past year?**

| Network members | Sent referrals to | Received referrals from | Exchange of patient information | Shared clinical care planning | Shared resources (i.e., co-funding for staff) |
|---|---|---|---|---|---|
| Organization A | | | | | |
| Organization B | | | | | |
| Organization C | | | | | |
| Organization D | | | | | |
| Organization E | | | | | |
| Organization F | | | | | |

4. **Relationship Quality: How would you describe the overall nature of your relationship with the following organizations in the past year based on the following categories?**

| Relationship quality | Description |
|---|---|
| **Not linked** | We did not work together (to serve patients with multiple chronic conditions) at all and have separate program goals. |
| **Communication** | We shared patient information only when it was advantageous to either or both programs. |
| **Cooperation** | We shared patient information and worked together when an opportunity arose. |
| **Coordination** | We worked side-by-side as separate organizations to achieve common program goals; efforts were coordinated to prevent overlap. |
| **Collaboration** | We worked side-by-side and actively pursued opportunities to work together to support patients with multiple chronic conditions, but did not establish a formal agreement. |
| **Partnership** | We worked together as a formal team with specified responsibilities to achieve common goals (had a Memorandum of Understanding or other formal agreement). |
| **Fully linked** | We mutually planned and shared staff and/or resources to organize and delivery care for individuals with multi-morbidity |

| Network members | Not linked | Communication | Coordination | Collaboration | Partnership | Fully linked |
|---|---|---|---|---|---|---|
| Organization A | | | | | | |
| Organization B | | | | | | |
| Organization C | | | | | | |
| Organization D | | | | | | |
| Organization E | | | | | | |
| Organization F | | | | | | |

5. **Benefits and drawbacks: Please select the benefits and drawbacks your organization has experienced in collaborating with other organizations to serve patients with multi-morbidity?**

| Impact of inter-organizational integration efforts | Already occurred | Expect to occur | Do not expect to occur |
|---|---|---|---|
| A. Benefits: | | | |
| 1) Improved capacity to better serve our patients with multi-morbidity in the community | | | |
| 2) Acquisition of additional funding or other resources to organize and deliver services | | | |
| 3) Acquisition of new knowledge or skills to better serve patients with multi-morbidity | | | |
| 4) Better use of our organization's services | | | |
| 5) Enhanced influence in the community | | | |
| 6) Other benefits (please describe) | | | |
| B. Drawbacks: | | | |
| 1) Takes too much time and resources | | | |
| 2) Loss of control/autonomy over decisions | | | |
| 3) Strained relations within my organization | | | |
| 4) Difficulty in dealing with other community partners/other members in the network | | | |
| 5) Not enough credit given to our organization | | | |
| 6) Other drawbacks (please describe) | | | |
