# Supplementary material for: Exploring Intra and Interorganizational Integration Efforts Involving the Primary Care Sector – A Case Study from Ontario
Source: Int J Integr Care. 2022 Sep 8;22(3):15. doi: 10.5334/ijic.5541 (PMC9461681; doi:10.5334/ijic.5541)
Supplement: Appendix 2. — Summary of network measures for two Health Links in Ontario (data collected in 2018). [file ijic-22-3-5541-s2.pdf]

Appendix 2: Summary of network measures for two Health Links in Ontario (data collected in 2018)

**Network metrics**	**Low-CPAT led case**	**High-CPAT led case**
**CONTACT**		
Density	0.65	0.49
Degree centralization	0.41	0.40
**PERCEIVED LEVEL OF INTEGRATION**		
Density	0.40	0.24
Degree centralization	0.36	0.47
**RELATIONSHIP TYPE**		
**REFERRALS (confirmed)**		
Density	0.12	0.27
Total Degree centralization (in and out degree)	0.36	0.44
In-degree centrality	0.26	0.50
Out-degree centrality	0.33	0.40
**INFORMATION SHARING**		
Density	0.23	0.500
Total Degree centralization (in and out degree)	0.56	0.49
In-degree centrality	0.29	0.25
Out-degree centrality	0.52	0.45
**JOINT CARE PLANNING**		
Density	0.28	0.33
Total Degree centralization (in and out degree)	0.50	0.69
In-degree centrality	0.39	0.43
Out-degree centrality	0.46	0.63
**SHARED RESOURCES**		
Density	0.10	0.02
Total Degree centralization (in and out degree)	0.38	0.08
In-degree centrality	0.28	0.07
Out-degree centrality	0.35	0.07
