# Supplementary material for: Exploring Intra and Interorganizational Integration Efforts Involving the Primary Care Sector – A Case Study from Ontario
Source: Int J Integr Care. 2022 Sep 8;22(3):15. doi: 10.5334/ijic.5541 (PMC9461681; doi:10.5334/ijic.5541)
Supplement: Appendix 3. — Member perceptions around the benefits and drawbacks of involvement in the Health Links program. [file ijic-22-3-5541-s3.pdf]

Appendix 3: Member perceptions around the benefits and drawbacks of involvement in the Health Links program

	Low-CPAT led case (n=14)			High-CPAT led case (n=11)		
**Perceived benefits**	**Already occurred**	**Expect to occur**	**Do not expect to occur**	**Already occurred**	**Expect to occur**	**Do not expect to occur**
Improved capacity to better serve our patients with multimorbidity in the community	57%	29%	14%	64%	27%	9%
Acquisition of additional funding or other resources to organize and deliver services	14%	43%	43%	18%	27%	55%
Obtained new knowledge or skills to better serve patients with multimorbidity	79%	21%	0%	64%	27%	9%
Improved use of our organization's services	71%	21%	7%	45%	45%	9%
Enhanced influence in the community	50%	36%	14%	36%	45%	18%
**Perceived drawbacks**	**Already occurred**	**Expect to occur**	**Do not expect to occur**	**Already occurred**	**Expect to occur**	**Do not expect to occur**
Takes too much time and resources	0%	14%	86%	36%	9%	55%
Loss of control/autonomy over decisions	0%	0%	100%	18%	0%	82%
Strained relations within my organization	0%	0%	100%	27%	9%	64%
Difficulty in dealing with other community partners/members in the network	0%	29%	71%	27%	18%	55%
Not enough credit given to our organization	7%	7%	86%	18%	27%	55%
